# Acknowledgement to Reviewers of *Bioengineering* in 2014

**DOI:** 10.3390/bioengineering2010001

**Published:** 2015-01-09

**Authors:** Bioengineering Editorial Office

**Affiliations:** MDPI AG Klybeckstrasse 64 CH-4057 Basel Switzerland

The editors of *Bioengineering* would like to express their sincere gratitude to the following reviewers for assessing manuscripts in 2014:

Brüser, Christoph
Centonze, Diego
Chew, Jia Wei
Choi, Yu Suk
Crawford, Neil R.
Cristofolini, Luca
Delvigne, Frank
Fischer, Rainer
Gawlitta, Debby
Georgiou, Melanie
Ghosh, Raja
Girão, Pedro Silva
Glassey, Jarka
Gomaa, Hassan
Hansmann, Jan
Hayes, Jessica
Hildebrandt, Martin
Hogwood, Cat
Huber, Gerd
Jaklenec, Ana
Jin, Li
Junne, Stefan
Kana, Evariste Bosco Gueguim
Kapsa, Robert M. I.
Keller, Thomas
Kleinsteuber, Sabine
Liddell, John
Liu, Ying
Lueders, Cora
Lyman, Philip
Monbouquette, Harold G.
Navarathne, Daminda
Ott, Denise
Park, Kwang Suk
Pedersen, Henrik
Puiggalí, Jordi
Qu, Haibin
Robles, Angel
Ronkainen, Niina J.
Saito, Kei
Salih, Vehid
Schnell, Sylvia
Schricker, Scott R.
Sell, Scott A.
Serino, Francesco
Sghir, Abdelghani
Sundararaghavan, Harini G.
Suzuki, Hiroaki
Takahashi, Hironobu
Tauer, Klaus
Tilles, Arno W.
Timmins, Nick
Tseng, Tina T.-C.
Van Dyke, Mark E.
Wurm, Florian
Yoshimoto, Makoto
Zhang, Hu
Zhang, Lijie Grace
Zhao, Feng

